# Investigating ocular ischemic events following pars plana vitrectomy

**DOI:** 10.1177/11206721251403004

**Published:** 2025-12-11

**Authors:** Ahmed M. Alshaikhsalama, Haafiz Hashim, David Shieh, Angeline L. Wang

**Affiliations:** 1 Department of Ophthalmology, 12334 UT Southwestern Medical Center, Dallas, TX, USA

**Keywords:** Retinal artery occlusion, retinal vein occlusion, non-arteritic ischemic optic neuropathy (NAION), pars plana vitrectomy.

## Abstract

**Objective:**

To assess the incidence of post–vitrectomy ocular ischemic events, including retinal artery occlusion (RAO), retinal vein occlusion (RVO), and non-arteritic ischemic optic neuropathy (NAION).

**Methods:**

This retrospective cohort study examined 120,350 patients who underwent vitrectomy. Using a multicenter healthcare database, patients with a CPT code for any vitrectomy procedure were assessed for subsequent ocular ischemic events. A follow-up analysis of 510,915 patients was performed to determine the risk attributable to vitrectomy versus clinical characteristics. Exclusion criteria included history of prior ocular ischemic events and less than 6 months of follow up.

**Results:**

The first post-operative month had a significantly higher risk of vascular occlusions compared to the 6–12-month period, with incidence rate ratios (IRRs) of 9.69 (95% CI: 6.9–13.7) for RAO, 11.43 (95% CI: 9.3–14.1) for RVO, and 8.75 (95% CI: 5.2–14.7) for NAION. The IRR for overall ischemic events was also increased during the first month following vitrectomy at 10.7 (95% CI: 9.0–12.7) and remained elevated up to 6 months later with an IRR of 1.49 (95% CI: 1.2–1.8) for the 3–6-month period compared to 6–12-months postoperatively. A separate multivariate analysis revealed that the risk of overall ischemic events was most correlated to vitrectomy (hazard ratio [HR] 3.32, 95% CI: 3.10–3.57) over multiple variables including cataract surgery (HR 1.49, 95% CI: 1.42–1.56) and nicotine use (HR 1.15, 95% CI: 1.08–1.22).

**Conclusions:**

Vitrectomy is correlated with significantly increased risk of ocular ischemic events, particularly within the first month following surgery. This risk gradually declines but remains elevated up to 6 months postoperatively.

## Introduction

Pars plana vitrectomy (PPV) is a surgical technique enabling access to the vitreous cavity and posterior segment.^1^ Approximately 225,000 vitrectomies are performed annually, and this number is expected to rise.^2^ Vitrectomy is indicated for a variety of vitreoretinal pathology, including vitreous hemorrhage, retinal detachment, and epiretinal membrane.^1^ Complications include spikes in intraocular pressure, corneal epithelial defects, and post-operative cataracts, as well as more serious sequelae such as endophthalmitis and sympathetic ophthalmia.^1,2^ These potential consequences warrant consideration in pre-, intra-, and post- operative management, including patient counseling during preoperative informed consent.^1^

Ocular ischemic events, especially retinal artery occlusion (RAO),^3–12^ have also been recognized as a potential complication of vitrectomy; however, their post-operative incidence remains poorly studied. Retinal vein occlusion (RVO) is the most common type of ocular ischemic event and has an estimated 15-year incidence of 2% in the general U.S. population.^13,14^ Non-arteritic anterior ischemic optic neuropathy (NAION) and retinal artery occlusion (RAO) are less common, with annual incidence rates of 6.9 per 100,000 and 1–2 per 100,000, respectively.^15,16^ Several mechanisms have been proposed explaining the association between vitrectomy and subsequent ocular ischemic events, including local anesthesia, elevated intra-operative intraocular pressure, compromised retinal perfusion, direct vessel injury, and underlying systemic disease.^5^ However, the relative rarity of this complication has limited existing research to case reports and small case series, preventing comprehensive analysis.^3–12^ This study seeks to address this gap by examining the association between vitrectomy and ocular ischemic events using population-level data from a multi-center national U.S. database. Specifically, this study aims to answer the following: 1. Is there an elevated risk for ocular ischemic events after vitrectomy? 2. When is the highest risk period following surgery? 3. How much does vitrectomy surgery increase the subsequent risk for a vascular event?

## Methods

This multi-center retrospective cohort study was conducted using an aggregated healthcare database, TriNetX, LLC, comprising data from 66 U.S. healthcare organizations with over 138 million patients. The database includes only de-identified information in the form of International Classification of Diseases Tenth revision (ICD-10) and current procedural terminology (CPT) codes. All information in the database is compliant with the Health Insurance Portability and Accountability Act (HIPAA) as well as ISO/IEC 27001:2013 certification standards. Given the de-identified, aggregate nature of the data, IRB approval was waived. We adhered to the Strengthening the Reporting of Observational Studies in Epidemiology (STROBE) guidelines in both study design and reporting.^17^ This study adhered to the principles of the Declaration of Helsinki.

### Cohort development:

The primary analysis followed a cohort of patients who underwent vitrectomy (CPT codes 67036, 67039, 67040, 67041, 67042, 67043, 67113 or 67108) for the development of incident ocular ischemic events; specifically, RAO (ICD-10 codes H34.1 and H34.2), RVO (ICD-10 codes H34.81 and H34.83), and NAION (ICD-10 code H47.01). Data in this study was collected on Aug. 26, 2025, from the U.S. Collaborative Network within the TriNetX database, including all patients records up to the collection date. Inclusion criteria for the primary analysis included age > 18 years and incidence of vitrectomy; the date of vitrectomy became the index date for the cohort. Exclusion criteria included prior history of RAO, RVO, NAION, giant cell arteritis (GCA), and less than six months of follow-up post-vitrectomy.

To assess the risk of ischemic events attributable to vitrectomy versus other factors, we also performed a secondary case-control analysis using Cox multivariate regression. We selected a separate cohort of patients with and without history of vitrectomy to serve as a reference population; inclusion criteria were age > 18 years and a history of cataracts (ICD-10 code H25) while the exclusion criteria was any history of RAO, RVO, NAION, or GCA. The selection of patients with an age-related cataract diagnosis for additional stratification was done to ensure all patients, including controls, received an ophthalmic examination as previously described.^18^ This cataract population was stratified by the presence of any type of vitrectomy procedure (CPT codes outlined previously). Cox regression was then performed, identifying the overall impact of different factors on ischemic event risk. Specifically, our analysis examined age, race, sex, and history of diabetes, hypertension, hyperlipidemia, nicotine dependence, glaucoma, cataract surgery, and vitrectomy. By including this secondary cohort analysis, we were able to assess the relative contribution of each covariate independently on vascular occlusion outcomes, which may be confounded in the vitrectomy-only cohort.

### Data collection

Baseline demographics such as age, sex, race, and systemic comorbidities were collected. New cases were recorded at 15 days post-operatively and then every month thereafter. Primary outcome measures included incident RAO, RVO, NAION, and any vascular occlusion that combined RAO/RVO/NAION outcomes. Our secondary analysis examined the relative contribution of baseline covariates on vascular occlusion risk.

### Statistical analysis

Statistical analysis was performed using Excel (Microsoft, 2024; WA, USA), PRISM (GraphPad 2024, Version 10; Boston, MA, USA), and built-in TriNetX analytics (TriNetX, LLC). To account for the difference in interval length for the first two intervals (0–15 days and 16–30 days, respectively) we calculated total incidence rate per interval using person-time analysis, recorded as events per 10,000 patient-months at risk. We then calculated the cumulative incidence rate of clinically meaningful intervals (0–1, 1–3, 3–6, 6–12, and 12–24 months) adjusted by patient-months; this approach standardizes risk estimates across intervals of different duration and avoids inflation of early postoperative risk due to shorter observation windows. Finally, we determined incidence rate ratios (IRRs) for each time interval relative to the 6–12 month reference period, using the standard error of the log (IRR) to calculate confidence limits. For the purposes of this analysis, vitrectomy was treated as a time-varying covariate, with ocular ischemic events only considered after the immediate index (vitrectomy) period up to 24 months.

Cox proportional hazards regression was also performed over a 1-year period for a multivariate analysis of outcome measures, assessed using the scaled Schoenfeld residuals approach.^19^ Patients with a history of age related cataracts were examined, considering the contribution of age, sex, race, history of vitrectomy or cataract surgery, systemic comorbidities, glaucoma, and smoking on ocular ischemic events. Age related cataract was selected as the baseline cohort for this analysis to ensure that all patients received an ophthalmic examination and to compare between cataract surgery and vitrectomy on outcome measures. Covariates may have occurred simultaneously and were not always present in each patient. Each covariate was treated as time-varying variable in the Cox model. Hazard ratios (HR) were calculated to assess the relative contribution to ocular ischemic event risk while holding other covariates constant, with time-to-event adjustments. Significance was considered at 2-sided p values <0.05.

A sub-analysis of the above analysis was performed stratifying the risk of subsequent vascular occlusion by individual vitrectomy CPT codes (67036, 67039, 67040, 67041, 67042, 67043, 67113, and 67108) as well as cataract surgery (66984). Each procedure type was modeled separately within the Cox framework, allowing the independent contribution of specific vitrectomy subtypes and cataract surgery to overall vascular occlusion risk to be assessed.

## Results

### Patient demographics

134,276 patients underwent vitrectomy during the study period. After applying inclusion/exclusion criteria, 120,350 patients were subsequently analyzed. The average age +/- standard deviation (SD) for the vitrectomy cohort was 60.1 +/- 17.0. The cohort contained slightly more males at 53.8%. Over one-third of patients had a chronic disease, with hypertension (54.2%), hyperlipidemia (36.0%) and diabetes mellitus (39.1%) being the most prevalent. Most patients (89.2%) were non-smokers at the time of surgery. Average BMI (mean +/- SD) was elevated above a normal range at 29.3 +/- 6.94. Characteristics of the vitrectomy cohort are summarized in Table 1.

**Table 1.** Baseline characteristics of patients with vitrectomy surgery.

Demographics	Vitrectomy Cohort (n = 120,350)
Age, years (Mean +/- SD)	60.1 +/- 17
*Gender (%)*	** **
Female	41,465 (44)
Male	50,998 (54)
Unknown Gender	2,297 (2)
*Race (%)*	
White	62,686 (66)
Black or African American	11,589 (12)
Hispanic/Latino	12,729 (13)
Asian	3,584 (4)
Unknown race	4,172 (5)
*Systemic Diseases (%)*	
Type 2 Diabetes mellitus	37,050 (39)
Obstructive Sleep Apnea	8,094 (0)
Nicotine Dependence	10,221 (11)
Hyperlipidemia	34,038 (36)
Hypertension	51,399 (54)
Chronic kidney disease	14,384 (15)
BMI (kg/m^2^) +/- SD	29.3 +/- 6.9

Abbreviations: BMI, body mass index.

### Incidence and relative risk of post-vitrectomy ocular ischemic events

**Figure 1.**
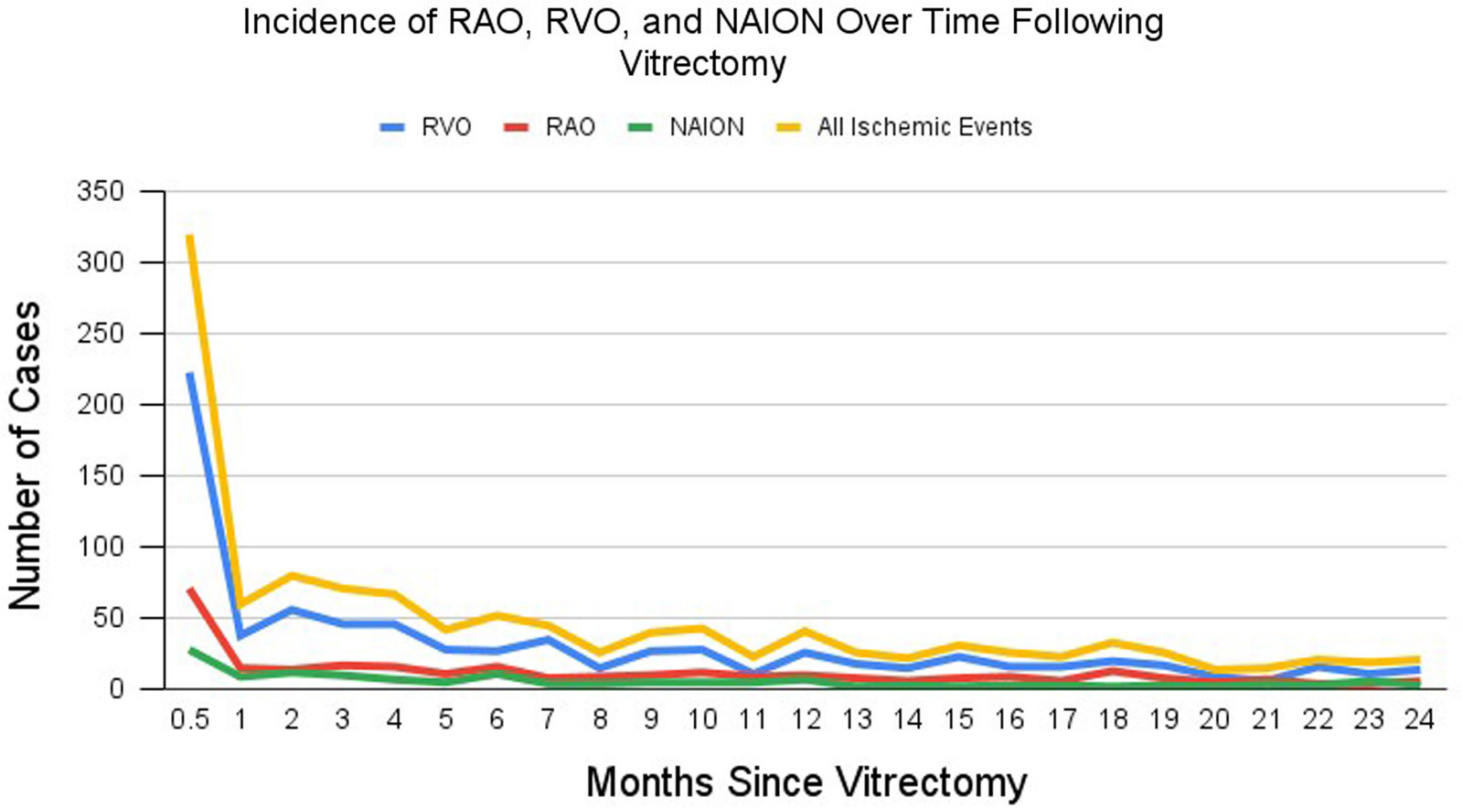
Shows the incidence of ocular ischemic events following vitrectomy over the two-year follow-up period. All time intervals were 1 month long except for the first two, which were 0–15 days and 15 days to 1 month, respectively. Abbreviations: RAO, retinal artery occlusion; RVO, retinal vein occlusion; NAION, non-arteritic ischemic optic neuropathy.

Figure 1 illustrates the total incidence of RAO, RVO, NAION, and any ocular ischemic event over the two-year follow up period following vitrectomy, with data shown in supplemental Table 1. The mean total follow-up for the vitrectomy cohort was 539.4 (SD: 275) days for the vitrectomy cohort. Incidence of RAO, RVO, NAION, and overall vascular occlusion following vitrectomy varied across postoperative intervals. Most ischemic events occurred in the immediate postoperative period, with 27.5% of all vascular events observed in the first 15 days (25.3% RAO, 29.1% RVO, and 22.0% NAION). In the 16–30-day window, incidence declined markedly, accounting for approximately 5% of RAO and RVO cases and approx. 6% of NAION cases. The 1–3 month and 3–6-month intervals each represented approximately 13–14% of total vascular events. By contrast, the 6–12-month period contributed only 18.3% of total events, compared with nearly 60% observed during the first 6 months. Between 12–24 months, a further decline was noted, with only 21.4% of cases recorded in that period. Cumulative two-year incidence was 277 RAO, 762 RVO, and 123 NAION cases, totaling 1,162 vascular occlusive events. At one year, the cumulative incidence per 10,000 patients was 17.8 for RAO, 50.5 for RVO, 7.97 for NAION, and 76.2 for all vascular events combined. Supplemental Table 2 shows the monthly rate of ocular ischemic events following vitrectomy per 10,000 vitrectomy surgeries, adjusted by duration following surgery.

**Figure 2.**
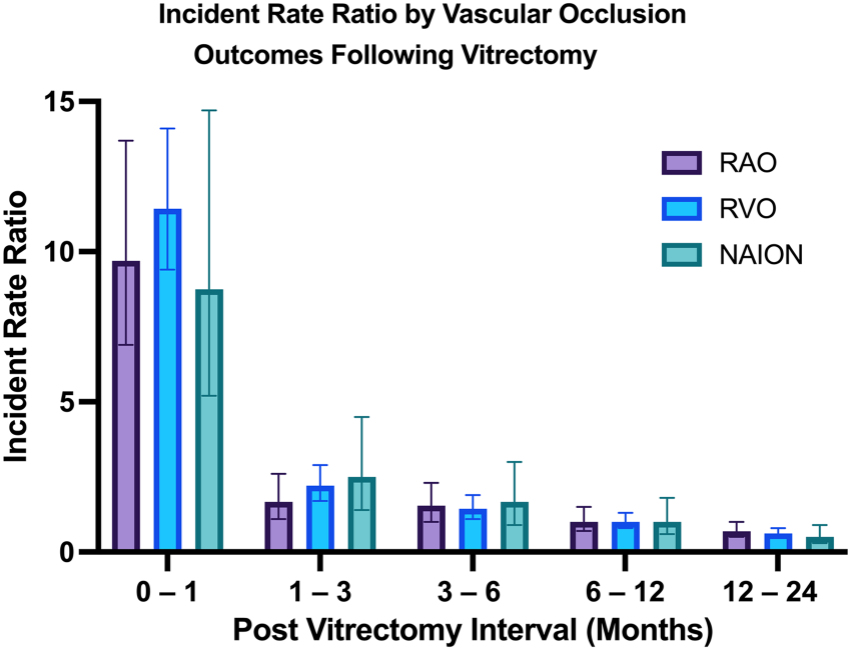
Shows the incident rate ratio of ocular ischemic events following vitrectomy across different time intervals compared to a 6–12 month interval over a two-year period. Abbreviations: RAO, retinal artery occlusion; RVO, retinal vein occlusion; NAION, non-arteritic ischemic optic neuropathy.

Table 2 shows incidence rate ratios (IRRs) per 10,000 patient-months for each postoperative interval compared with the 6–12-month reference period. The first postoperative month carried the highest risk, with an overall IRR of 10.7 (95% CI: 9.0–12.7). Early risk elevation was consistent across subtypes: RAO IRR 9.7 (95% CI: 6.9–13.7), RVO IRR 11.4 (95% CI: 9.3–14.1), and NAION IRR 8.8 (95% CI: 5.2–14.7). Risk declined but remained significantly elevated at 1–3 months postoperatively (overall IRR 2.1, 95% CI: 1.7–2.6) and 3–6 months (IRR 1.5, 95% CI: 1.2–1.8). During the 12–24-month period, incidence fell below the reference interval, with an overall IRR of 0.63 (95% CI: 0.5–0.7). These results support that ischemic events are most likely to occur in the immediate postoperative period; risk remains elevated up to the first six months across all ischemic event types. Figure 2 illustrates the IRRs of RAO, RVO, and NAION (compared to 6–12 months) over the two-year follow up period following vitrectomy.

**Table 2.** Incident rate ratios (IRR) of ocular ischemic events post-vitrectomy compared with the 6–12 month reference interval. Rates calculated per 10,000 patient-months.

Time Period After Vitrectomy	IRR of RAO (95% CI)	IRR of RVO (95% CI)	IRR of NAION (95% CI)	IRR of Overall Vascular Occlusion (95% CI)
0–1 month	**9.69** (**6.9–13.7)**	**11.43** (**9.3–14.1)**	**8.75** (**5.2–14.7)**	**10.7** (**9.0–12.7)**
1–3 months	**1.67** (**1.1–2.6)**	**2.21** (**1.7–2.9)**	**2.5** (**1.4–4.5)**	**2.11** (**1.7–2.6)**
3–6 months	**1.54** (**1.0–2.3)**	**1.44** (**1.1–1.9)**	1.67 (0.9–3.0)	**1.49** (**1.2–1.8)**
6–12 months	1.0 (0.7–1.5)	1.0 (0.8–1.3)	1.0 (0.6–1.8)	1.0 (0.8–1.2)
12–24 months	0.69 (0.5–1.0)	**0.62** (**0.5–0.8)**	**0.5** (**0.3–0.9)**	**0.63** (**0.5–0.7)**

Abbreviations: RAO, retinal artery occlusion; RVO, retinal vein occlusion; NAION, non-arteritic ischemic optic neuropathy; CI: Confidence interval. Significant values in bold (p < 0.05).

### Multivariate assessment of post-vitrectomy ocular ischemic events

A total of 510,915 patients were included in the multivariate analysis of ocular ischemic event risk with their baseline characteristics shown in Table 3. Of these, 49,285 patients had undergone vitrectomy. Table 4 summarizes the results of the Cox proportional hazards analysis, which examined the contribution of multiple covariates on ocular ischemic event risk in patients with a history of age-related cataracts. Ocular surgery was the strongest predictor of ocular ischemic events across all categories, with a history of vitrectomy contributing HRs of 3.34 (95% CI: 2.93–3.82) for RAO, 3.67 (95% CI: 3.36–4.01) for RVO, 2.16 (95% CI: 1.78–2.61) for NAION, and 3.32 (95% CI: 3.10–3.57) for all ocular ischemic events. This was followed by a history of cataract surgery, with HRs of 1.52 for RAO, 1.48 for RVO, 1.57 for NAION, and 1.49 for all occlusions. Systemic comorbidities also influenced ocular ischemic event risk: Type 2 diabetes mellitus resulted in HRs of 1.34 for RAO, 1.18 for RVO, 1.25 for NAION, and 1.23 for overall ocular ischemic events, with similar, albeit weaker, associations observed with essential hypertension. Glaucoma also significantly increased risk, with HRs of 1.13 for RAO, 1.61 for RVO, 1.16 for NAION, and 1.40 for overall ocular ischemic events. These findings suggest ocular surgery, specifically vitrectomy, may contribute to ocular ischemic events to a greater degree than other measured factors. Covariates with HRs below 1.1 were considered less impactful.

**Table 3.** Baseline characteristics of secondary analysis cohort

Demographics	Cataract Cohort (n = 510,915)
Age, years (Mean +/- SD)	66.1 ± 10.1
*Gender (%)*	** **
Female	281,003 (55)
Male	209,475 (41)
Unknown Gender	20,437 (4)
*Race (%)*	
White	342,313 (67)
Black or African American	71,528 (14)
Hispanic/Latino	35,764 (7)
Asian	20,437 (4)
Unknown race	40,403 (8)
*Systemic Diseases (%)*	
Type 2 Diabetes mellitus	255,458 (50)
Obstructive Sleep Apnea	183,929 (36)
Nicotine Dependence	45,982 (9)
Hyperlipidemia	51,092 (10)
Hypertension	137,947 (27)
Chronic kidney disease	45,982 (9)
BMI (kg/m^2^) +/- SD	29.5 +/- 6.9
Any Incident Vitrectomy	49, 285 (10)

Abbreviations: BMI, body mass index.

**Table 4.** Cox proportional hazards ratio assessing the relative contribution of covariates on ocular ischemic events.

Covariates	RAO Hazards Ratio (95% CI)	P value	RVO Hazards Ratio (95% CI)	P value	NAION Hazards Ratio (95% CI)	P value	Overall Vascular Occlusion Hazards Ratio (95% CI)	P value
**Vitrectomy**	3.34 (2.93, 3.82)	< 0.001	3.67 (3.36, 4.01)	< 0.001	2.16 (1.78, 2.61)	< 0.001	3.32 (3.10, 3.57)	< 0.001
Cataract Surgery	1.52 (1.38, 1.66)	< 0.001	1.48 (1.39, 1.57)	< 0.001	1.57 (1.40, 1.77)	< 0.001	1.49 (1.42, 1.56)	< 0.001
Glaucoma	1.13 (1.04, 1.23)	0.005	1.61 (1.53, 1.70)	< 0.0001	1.16 (1.05, 1.30)	0.0058	1.40 (1.35, 1.47)	< 0.001
Male	1.34 (1.24, 1.44)	< 0.001	1.17 (1.11, 1.23)	< 0.001	1.49 (1.36, 1.63)	< 0.001	1.26 (1.21, 1.31)	< 0.001
Type 2 Diabetes Mellitus	1.34 (1.23, 1.45)	< 0.001	1.18 (1.12, 1.25)	< 0.0001	1.25 (1.13, 1.39)	< 0.001	1.23 (1.18, 1.29)	< 0.001
Hypertension	1.30 (1.19, 1.42)	< 0.001	1.15 (1.08, 1.22)	< 0.001	1.12 (1.00, 1.25)	0.040	1.17 (1.12, 1.23)	< 0.001
Nicotine Dependence	1.48 (1.34, 1.65)	< 0.001	1.06 (0.97, 1.15)	0.20	0.99 (0.85, 1.16)	0.91	1.15 (1.08, 1.22)	< 0.001
Age at Index	1.03 (1.02, 1.03)	< 0.001	1.04 (1.04, 1.04)	< 0.001	1.01 (1.00, 1.01)	0.001	1.03 (1.03, 1.03)	< 0.001
Hyperlipidemia	1.13 (1.04, 1.23)	0.0029	0.94 (0.89, 0.99)	0.0221	0.90 (0.81, 1.00)	0.052	0.98 (0.94, 1.03)	0.43
Black or African American	0.79 (0.69, 0.90)	0.006	1.02 (0.94, 1.11)	0.63	0.82 (0.69, 0.97)	0.020	0.93 (0.87, 0.99)	0.034
Hispanic or Latino	0.62 (0.53, 0.75)	< 0.001	0.91 (0.82, 1.01)	0.084	0.68 (0.56, 0.84)	0.0004	0.79 (0.73, 0.86)	< 0.001
Asian	0.55 (0.43, 0.70)	< 0.001	0.84 (0.73, 0.96)	0.011	0.26 (0.17, 0.40)	< 0.001	0.68 (0.60, 0.76)	< 0.001
White	0.83 (0.75, 0.92)	0.002	0.74 (0.69, 0.79)	< 0.001	0.82 (0.73, 0.93)	0.0018	0.78 (0.74, 0.82)	< 0.001

Abbreviations: RAO, retinal artery occlusion; RVO, retinal vein occlusion; NAION, non-arteritic ischemic optic neuropathy; CI: Confidence interval.

Table 5 shows a sub analysis of the above Cox proportional hazards model. This analysis compared the effect of different vitrectomy subtypes on ischemic event risk compared to the risk of the vitrectomy group as a whole. Of note, this analysis only compares vitrectomy subtypes to each other without assessing their risk compared to non-vitrectomy patients or other comorbidities and demographic variables (as shown in Table 4); thus, vitrectomy subtypes with hazard ratios less than one may still be associated with increased overall risk of ocular ischemic event. When stratifying vitrectomy by CPT code, retinal detachment repair (CPT 67108) was the strongest risk factor for subsequent occlusion, with HRs of 4.10 (95% CI: 3.21–5.22) for RAO, 3.71 (95% CI: 3.19–4.33) for RVO, 2.18 (95% CI: 1.59–3.01) for NAION, and 3.45 (95% CI: 3.06–3.89) for overall vascular occlusion risk. Other procedures had weaker effects, though panretinal photocoagulation (67040) was notably associated with an increased risk of subsequent RVO HR 1.88 (95% CI: 1.31–2.72). Cataract surgery (66984) also showed lower HRs relative to vitrectomy procedures as a whole, consistent with our risk comparison of vitrectomy and cataract surgery from Table 4. Demographic factors also played a role, with Male sex (HRs 1.17–1.60) consistently associated with increased risk. Overall, surgical retinal detachment repair demonstrated the strongest association with subsequent ischemic events, with other vitrectomy subtypes showing more limited risk.

**Table 5.** Cox proportional hazards ratio assessing the relative contribution of vitrectomy CPT codes on ocular ischemic events.

Covariates	RAO HR (95% CI)	P value	RVO HR (95% CI)	P value	NAION HR (95% CI)	P value	Overall Vascular Occlusion HR (95% CI)	P value
67108 – RD repair	4.10 (3.21, 5.22)	<0.01	3.71 (3.19, 4.33)	<0.01	2.18 (1.59, 3.01)	<0.01	3.45 (3.06, 3.89)	<0.01
67113 – Complex RD repair	0.78 (0.38, 1.60)	0.50	1.44 (1.02, 2.04)	0.04	0.94 (0.44, 2.02)	0.87	1.19 (0.89, 1.58)	0.24
67036 – PPV	1.18 (0.63, 2.21)	0.60	1.31 (0.93, 1.86)	0.13	0.78 (0.31, 1.93)	0.59	1.14 (0.85, 1.53)	0.37
67039 – PPV + focal Endolaser	1.33 (0.48, 3.63)	0.58	0.76 (0.36, 1.62)	0.48	1.02 (0.25, 4.16)	0.98	1.00 (0.58, 1.70)	0.99
67041 – ERM removal	0.23 (0.03, 1.64)	0.14	1.03 (0.61, 1.74)	0.91	0.62 (0.15, 2.55)	0.51	0.94 (0.60, 1.46)	0.78
67042 – ILM peel	0.98 (0.40, 2.38)	0.96	1.42 (0.95, 2.14)	0.09	1.29 (0.52, 3.15)	0.58	1.40 (1.01, 1.94)	0.05
67043 – Subretinal membrane	1.90 (0.26, 13.77)	0.53	1.19 (0.29, 4.81)	0.81	N/A*	0.96	1.17 (0.37, 3.65)	0.79
67040 – PRP (endolaser)	1.58 (0.83, 3.03)	0.17	1.88 (1.31, 2.72)	<0.01	0.80 (0.29, 2.18)	0.66	1.48 (1.08, 2.02)	0.01
66984 – Cataract surgery	0.70 (0.50, 0.99)	0.04	0.80 (0.66, 0.96)	0.02	0.93 (0.66, 1.32)	0.67	0.81 (0.70, 0.94)	0.00

Abbreviations: PPV, pars plana vitrectomy; RD, retinal detachment; PRP, panretinal photocoagulation; ERM, epiretinal membrane; ILM, internal limiting membrane; RAO, retinal artery occlusion; RVO, retinal vein occlusion; NAION, non-arteritic ischemic optic neuropathy; CI: Confidence interval.

*Estimate unable due to sparse events

## Discussion

Ocular ischemic events have been recognized as a possible complication of vitrectomy in recent years.^3–12^ This retrospective cohort study aimed to characterize this relationship on a population level. We identified a time-dependent association between ocular ischemic events and vitrectomy, with nearly one-third of total events over the first two post-operative years occurring in the first month following surgery. Incident rate comparisons corroborated this relationship, revealing an 10-fold increased risk of developing any ocular ischemic event within the first month compared to the 6–12 month time interval following vitrectomy. A separate multivariate analysis found vitrectomy to be the most highly correlated risk factor for ocular ischemic events in patients with a history of cataracts. Thus, our data indicates an association between vitrectomy and subsequent ocular ischemic events, suggesting that RAO, RVO, and NAION may be rare complications of vitrectomy.

This study found a higher-than-expected incidence of ocular ischemic events following vitrectomy. Over the two-year observation period, our analysis observed a total of 1162 total ocular ischemic events out of 120,350 patients, a total incidence of approximately 1%. When accounting for time of analysis, we see an overall incidence of nearly 50 per 10,000 patients undergoing vitrectomy per year. Comparing this to epidemiological studies of ocular ischemic events, we observed post-vitrectomy incidence rates are much higher than in the general population. An early case series by Rumelt et al found an average RAO incidence of 1 per 100,000 patients per year, over 100-fold times lower than the rate observed post-vitrectomy in this study.^20^ Similarly, a meta-analysis examining the incidence of RVO in the general population observed a pooled yearly incidence of 163 patients per 100,000 annually, roughly half the rate seen in this analysis.^21^ For NAION, the estimated incidence is generally thought to be between 2 and 10 per 100,000 patients annually, which is at most one-fifth of the risk observed in this investigation.^22–24^ While some of this increase in ischemic event incidence may be attributed to retinal disease and comorbidities more common in the vitrectomy patient population, the magnitude of increase in the incidence rate suggests that vitrectomy may potentially be a risk factor for ocular ischemic events.

We also observed that ocular ischemic event risk peaked in the first month after vitrectomy. This is concordant with smaller investigations identifying the first post-operative month as a high-risk period. One case series by Fischer et al found that at least 2/3 of post-vitrectomy vascular occlusions occurred within the first month.^5^ This was further observed in a seperate case series by Tappeiner et al, which reported 6 patients presenting with RAO 2–14 days following vitrectomy.^12^ In some cases, vascular occlusion can occur even sooner, as observed by Ellaban et al in a case where a patient experienced an intra-operative central RAO.^3^

Several explanations have been proposed for the rise in vascular occlusion risk. Both Fischer and Tappeiner attributed anesthesia as a potential cause; peribulbar anesthesia has been associated with vasospasm and decreased retinal blood flow,^5,12,25^ while endotracheal intubation has been linked to increased intraocular pressure and decreased ocular perfusion as a stress response.^5^ Another possible factor is gas endotamponade, which may cause immediate postoperative IOP spikes due to gas overfill or expansion, especially with expansile gas mixtures such as >14% C3F8 or >20% SF_6_.^5^ Gas-mediated increases in IOP can often be delayed, as seen in an analysis by Thompson et al where peak IOP was measured between 2 days and 2 weeks after macular hole surgery.^26^ Another factor is poor ocular perfusion; Okamoto et al observed a significant decrease in ocular blood flow during vitrectomy, particularly at higher infusion pressures.^27^ This drop in perfusion may also be related to the underlying surgical indication and procedure; an analysis by Iwase et al using laser speckle flowgraphy showed decreased retinal perfusion in patients with rhegmatogenous retinal detachment that returned to baseline following vitrectomy with scleral buckling.^28^ This variety of potential mechanisms highlights the complex interplay of factors influencing ocular ischemic event risk during and following vitrectomy, reinforcing the need for individualized risk assessment and risk stratification by type of vitrectomy procedure.

Furthermore, multivariate analysis revealed vitrectomy was the strongest potential contributor to ocular ischemic event risk, with a 2 to 3-fold increased HR after adjusting for other covariates. This risk surpassed even the potential contribution of cataract surgery, which has been long documented as a potential risk factor for ocular ischemic events.^29–34^ A multicenter study using information from the Intelligent Research in Sight (IRIS) registry found that cataract surgery increased the risk of central RVO in the following year by approximately 25%, while a meta-analysis by Shew et al found that the risk of NAION increased fourfold in the year following cataract surgery.^30,31^ Similarly, a case series by Sen et al describes 14 patients with RAO who presented within 4 days of cataract surgery.^29^ Despite this known association, our study found that vitrectomy posed more than double the risk of ocular ischemic events compared to cataract surgery (HR 3.32; 95% CI, 3.10–3.57 vs. 1.49; 95% CI, 1.42–1.56). Additionally, vitrectomy demonstrated a stronger association with ocular ischemic events than advanced age, hypertension, diabetes, and nicotine dependence, all of which are risk factors previously identified in the literature.^35–37^ For instance, a retrospective evaluation of 33 patients by Rudkin et al identified hypertension, hyperlipidemia, and prior vascular disease as central RAO risk factors.^35^ Moreover, the Beaver Dam Eye Study, which longitudinally examined approximately 5,000 patients with RVO, found an association with hypertension, diabetes, and smoking.^36^ Similarly, a literature review on NAION by Kerr et al cited diabetes, hypertension, and hyperlipidemia as potential contributors to NAION.^37^ Our study found a significant association between vitrectomy and all types of ocular ischemic event, surpassing the impact of previously identified risk factors even after adjustment with multivariate analysis.

Despite the significant association observed between vitrectomy and ocular ischemic events, this study has several caveats. First, the overall incidence of post-vitrectomy ischemic events remains quite low: Approximately 50 per 10,000 vitrectomy procedures per year. However, this is comparable to other vision-threatening complications such as endophthalmitis, which has an estimated incidence rate of 3–18 per 10,000.^38,39^ Furthermore, ischemic events may have widely variable effects on vision; depending on the location and severity, patients’ visual acuity can be severely impaired or almost unaffected.^40,41^ Considering that vitrectomies are often performed due to vision-threatening indications, it is important to balance the risks of ocular ischemic events with the potential risk of not undergoing the procedure. Ultimately, the decision to undergo or defer such a potentially vision altering procedure involves a risk-benefit discussion between the patient and physician; the hope of the authors is that this analysis provides data and context to aid in this endeavor.

This study has several strengths. A key strength is its large, heterogenous U.S. patient population. To our knowledge, this is the largest cohort study to date analyzing vitrectomy and subsequent ocular ischemic event risk, enabling better examination of this potential relationship. Another major strength is the access to longitudinal patient data through TriNetX, allowing examination of patient data up to two years post-operatively. Finally, our investigation's multivariate analysis enabled us to account for potential confounding variables and compare the relative contribution of vitrectomy to ocular ischemic event risk.

This study also has several limitations. Firstly, the retrospective study design, despite the strength and time dependent associations observed, limit conclusions regarding causality of the underlying relationship. In the same vein is the risk of confounding by indication; in two patients with the same disease (e.g., retinal detachment), one may have more severe disease which may require vitrectomy. Reliance on ICD-10 codes limits our ability to capture disease severity, which could potentially be a major confounder; this is especially apparent for surgical indications such as retinal detachment or NAION, which often have a wide range of locations, presentations, and etiologies. Consequently, subsequent vascular occlusions may be due to the increased severity of the underlying disease, not the vitrectomy itself. As an attempt to mitigate this potential bias, we have incorporated a multivariate secondary analysis, which adjusts risk based on baseline comorbidities; however, this analysis does not account for disease severity. Furthermore, this secondary analysis utilized the TriNetX's Cox proportional hazards analysis model, which does not provide data about individual covariates, such as the number of individual vitrectomy sub procedures or numbers of each covariate utilized in the analysis. Consequently, sample size and power-issues may affect the results of these analyses (Table 4 and 5) and may over or underestimate the attributed risk between covariates and subsequent ischemic event risk. Additionally, this study does not account for eye laterality since outcomes were considered per individual. Furthermore, while we were able to exclude individuals with a prior history of ocular ischemic events, some patients may have had prior clinically silent occlusions which were detected only during routine post-operative monitoring, thereby overinflating vascular occlusion data in the early postoperative months. The observed results may also be susceptible to detection bias, as patients could have been more likely to attend follow-up appointments within the first month after surgery, increasing the likelihood of capturing ischemic events and artificially inflating the temporality and strength of the observed associations. Additionally, patients may have transitioned to other ophthalmologists or healthcare systems, potentially leading to under detection of ocular ischemic events occurring after six months of surgery. Ultimately, large-scale prospective randomized clinical trials are needed to definitively determine the exact relationship between vitrectomy and ocular ischemic events, including stratification by age, disease severity, surgical indication, and medication supplement use. However, this study supports the need for additional investigation into ocular ischemic events as a potential risk of vitrectomy.

In conclusion, this study found a potential relationship between vitrectomy and subsequent ocular ischemic events that was most pronounced within the first month following surgery. Given the strength and temporality of this association, we propose that RAO, RVO, and NAION should be considered as possible rare post operative complications of vitrectomy. Further studies are required to explore the underlying pathophysiology, establish causation, and identify methods to mitigate the risk of these sight-threatening complications.

## Supplemental Material

sj-docx-1-ejo-10.1177_11206721251403004 - Supplemental material for Investigating ocular ischemic events following pars plana vitrectomy Supplemental material, sj-docx-1-ejo-10.1177_11206721251403004 for Investigating ocular ischemic events following pars plana vitrectomy by Ahmed M. Alshaikhsalama, Haafiz Hashim, David Shieh and Angeline L. Wang in European Journal of Ophthalmology

sj-docx-2-ejo-10.1177_11206721251403004 - Supplemental material for Investigating ocular ischemic events following pars plana vitrectomy Supplemental material, sj-docx-2-ejo-10.1177_11206721251403004 for Investigating ocular ischemic events following pars plana vitrectomy by Ahmed M. Alshaikhsalama, Haafiz Hashim, David Shieh and Angeline L. Wang in European Journal of Ophthalmology

## References

[1] Omari A Mahmoud TH . Vitrectomy. *StatPearls*. StatPearls Publishing Copyright © 2024, StatPearls Publishing LLC.; 2024.

[2] Lalezary M Shah RJ Reddy RK , et al. Prospective retinal and optic nerve vitrectomy evaluation (PROVE) study: twelve-month findings. Ophthalmology Oct 2014; 121: 1983–1989. doi:10.1016/j.ophtha.2014.04.008 24907063

[3] Ellabban AA Patil AD Costen MT , et al. Central retinal artery occlusion during vitrectomy: immediate retinal revascularization following induction of posterior vitreous detachment. Am J Ophthalmol Case Rep Mar 2018; 9: 38–40. doi:10.1016/j.ajoc.2018.01.008 29468216 PMC5786868

[4] Malinowski SM Pesin SR . Visual field loss caused by retinal vascular occlusion after vitrectomy surgery. Am J Ophthalmol May 1997; 123: 707–708. doi:10.1016/s0002-9394(14)71093-1 9152086

[5] Fischer C Bruggemann A Hager A , et al. Vascular occlusions following ocular surgical procedures: a clinical observation of vascular complications after ocular surgery. J Ophthalmol 2017; 2017:: 9120892. doi:10.1155/2017/9120892 28781891 PMC5525065

[6] Fang IM Huang JS . Central retinal artery occlusion caused by expansion of intraocular gas at high altitude. Am J Ophthalmol Oct 2002; 134: 603–605. doi:10.1016/s0002-9394(02)01631-8 12383821

[7] Liu G Zhang J Huang X , et al. Revascularization central retinal artery occlusion during vitrectomy within 5 minutes. Asia-Pacific Journal of Ophthalmology 2022/09/01/ 2022; 11: 491. doi:10.1097/APO.0000000000000451 35342174

[8] Murao F Kinoshita T Katome T , et al. Suspected gentamicin-induced retinal vascular occlusion after vitrectomy. Case Rep Ophthalmol May-Aug 2020; 11: 473–480. doi:10.1159/000509337 32999678 PMC7506252

[9] Russell JF Scott NL Haddock LJ , et al. Central retinal artery occlusion on postoperative day one after vitreoretinal surgery. Am J Ophthalmol Case Rep Dec 2018; 12: 93–96. doi:10.1016/j.ajoc.2018.10.001 30364763 PMC6197795

[10] Saito W Yamamoto S Takeuchi S , et al. Ophthalmic artery occlusion following pars plana vitrectomy in a patient with terson's syndrome. Br J Ophthalmol Sep 2002; 86: 1063–1064. doi:10.1136/bjo.86.9.1063-a PMC1771289 12185139

[11] Stavrakas P Karmiris E Tranos P , et al. Paracentral acute middle maculopathy following surgically induced branch retinal artery occlusion during vitrectomy. Case Rep Ophthalmol 2021; 12: 25–31. doi:10.1159/000510558 33613247 PMC7879272

[12] Tappeiner C Garweg JG . Retinal vascular occlusion after vitrectomy with retrobulbar anesthesia-observational case series and survey of literature. Graefes Arch Clin Exp Ophthalmol Dec 2011; 249: 1831–1835. doi:10.1007/s00417-011-1783-9 21850439

[13] Laouri M Chen E Looman M , et al. The burden of disease of retinal vein occlusion: review of the literature. Eye 2011-08 2011; 25: 981–988. doi:10.1038/eye.2011.92 21546916 PMC3178209

[14] Blair K Czyz CN . Central Retinal Vein Occlusion. StatPearls. StatPearls Publishing, 2024. 30252241

[15] Mansukhani SA Chen JJ Fairbanks AM , et al. A population-based study of anterior ischemic optic neuropathy following cataract surgery. Am J Ophthalmol. Feb 2021; 222: 157–165. doi:10.1016/j.ajo.2020.08.020 32818451 PMC7889757

[16] Ho K-Y Lin C-D Hsu T-J , et al. Increased risks of retinal vascular occlusion in patients with migraine and the protective effects of migraine treatment: a population-based retrospective cohort study. Sci Rep 2024/07/04 2024; 14: 15429. doi:10.1038/s41598-024-66363-9 38965381 PMC11224338

[17] Vandenbroucke JP von Elm E Altman DG , et al. Strengthening the reporting of observational studies in epidemiology (STROBE): explanation and elaboration. PLoS Med 2007; 4: e297. doi:10.1371/journal.pmed.0040297 PMC2020496 17941715

[18] Alshaikhsalama AM Alsoudi AF Wai KM , et al. Association between Obstructive Sleep Apnea and Age-related Macular Degeneration Development and Progression. Ophthalmol Retina Dec 9 2024. doi:10.1016/j.oret.2024.12.004 39662591

[19] GRAMBSCH PM THERNEAU TM . Proportional hazards tests and diagnostics based on weighted residuals. Biometrika 1994; 81: 515–526. doi:10.1093/biomet/81.3.515

[20] Rumelt S Dorenboim Y Rehany U . Aggressive systematic treatment for central retinal artery occlusion. Am J Ophthalmol 1999/12/01/ 1999; 128: 733–738. doi:10.1016/S0002-9394(99)00359-1 10612510

[21] Song P Xu Y Zha M , et al. Global epidemiology of retinal vein occlusion: a systematic review and meta-analysis of prevalence, incidence, and risk factors. J Glob Health. Jun 2019; 9: 010427. doi:10.7189/jogh.09.010427 PMC6513508 31131101

[22] Hathaway JT Shah MP Hathaway DB , et al. Risk of nonarteritic anterior ischemic optic neuropathy in patients prescribed semaglutide. JAMA Ophthalmol 2024; 142: 732–739. doi:10.1001/jamaophthalmol.2024.2296 38958939 PMC11223051

[23] Johnson LN Arnold AC . Incidence of nonarteritic and arteritic anterior ischemic optic neuropathy. Population-based study in the state of Missouri and Los Angeles county, California. J Neuroophthalmol Mar 1994; 14: 38–44. 8032479

[24] Hattenhauer MG Leavitt JA Hodge DO , et al. Incidence of nonarteritic anterior ischemic optic neuropathy. Am J Ophthalmol Jan 1997; 123: 103–107. doi:10.1016/s0002-9394(14)70999-7 9186104

[25] Findl O Dallinger S Menapace R , et al. Effects of peribulbar anesthesia on ocular blood flow in patients undergoing cataract surgery. Am J Ophthalmol Jun 1999; 127: 645–649. doi:10.1016/s0002-9394(99)00066-5 10372873

[26] Thompson JT Sjaarda RN Glaser BM , et al. Increased intraocular pressure after macular hole surgery. Am J Ophthalmol 1996; 121: 615–622. doi:10.1016/S0002-9394(14)70626-9 8644803

[27] Okamoto M Matsuura T Ogata N . Ocular blood flow before, during, and after vitrectomy determined by laser speckle flowgraphy. Ophthalmic Surg Lasers Imaging Retina. Mar-Apr 2014; 45: 118–124. doi:10.3928/23258160-20140306-04 24635152

[28] Iwase T Kobayashi M Yamamoto K , et al. Changes in blood flow on optic nerve head after vitrectomy for rhegmatogenous retinal detachment. Invest Ophthalmol Vis Sci Nov 1 2016; 57: 6223–6233. doi:10.1167/iovs.16-20577 27842162

[29] Sen A Mitra A Tripathi S , et al. A cluster of central retinal artery occlusions following cataract surgery. Indian J Ophthalmol May 2019; 67: 630–633. doi:10.4103/ijo.IJO_1070_18 31007223 PMC6498933

[30] Bagdasarova Y Lee AY Maring M , et al. Cataract surgery is not associated with decreased risk of retinal vein occlusion. Ophthalmol Sci Sep 2021; 1: 100041. doi:10.1016/j.xops.2021.100041 36275940 PMC9562376

[31] Shew W Wang MTM Danesh-Meyer HV . Nonarteritic anterior ischemic optic neuropathy after cataract surgery: a systematic review and meta-analysis. J Neuroophthalmol. Mar 1 2023; 43: 17–28. doi:10.1097/wno.0000000000001625 36166807

[32] Yusuf IH Fung TH Wasik M , et al. Transient retinal artery occlusion during phacoemulsification cataract surgery. Eye 2014/11/01 2014; 28: 1375–1379. doi:10.1038/eye.2014.190 25104741 PMC4274288

[33] Wang LA Yang AS Su YC , et al. Cataract surgery and incidence of retinal vascular occlusion: population-based cohort study using a target trial emulation framework. Am J Ophthalmol Dec 2024; 268: 143–154. doi:10.1016/j.ajo.2024.07.029 39097255

[34] Deshmukh R Narula R . Commentary: central retinal arterial occlusions after phacoemulsification: our perspective. Indian J Ophthalmol 2019; 67: 633–634. doi:10.4103/ijo.IJO_280_19 31007224 PMC6498923

[35] Rudkin AK Lee AW Chen CS . Vascular risk factors for central retinal artery occlusion. Eye 2010/04/01 2010; 24: 678–681. doi:10.1038/eye.2009.142 19521436

[36] Klein R Klein BE Moss SE , et al. The epidemiology of retinal vein occlusion: the beaver dam eye study. Trans Am Ophthalmol Soc 2000; 98: 133–141. discussion 141-3. 11190017 PMC1298220

[37] Kerr NM Chew SSSL Danesh-Meyer HV . Non-arteritic anterior ischaemic optic neuropathy: a review and update. J Clin Neurosci 2009/08/01/ 2009; 16: 994–1000. doi:10.1016/j.jocn.2009.04.002 19596112

[38] Chen G Tzekov R Li W , et al. INCIDENCE OF ENDOPHTHALMITIS AFTER VITRECTOMY: a systematic review and meta-analysis. Retina May 2019; 39: 844–852. doi:10.1097/iae.0000000000002055 29370034

[39] Hosseini S Daraee G Shoeibi N , et al. Incidence rate and clinical characteristics of acute endophthalmitis following 23-gauge pars plana vitrectomy. Int J Retina Vitreous Dec 21 2022; 8: 85. doi:10.1186/s40942-022-00435-8 36544227 PMC9768931

[40] Mirshahi A Feltgen N Hansen LL , et al. Retinal vascular occlusions: an interdisciplinary challenge. Dtsch Arztebl Int. Jun 2008; 105: 474–479. doi:10.3238/arztebl.2008.0474 19626196 PMC2696914

[41] Scott IU Ip MS VanVeldhuisen PC , et al. A randomized trial comparing the efficacy and safety of intravitreal triamcinolone with standard care to treat vision loss associated with macular edema secondary to branch retinal vein occlusion: the standard care vs corticosteroid for retinal vein occlusion (SCORE) study report 6. Arch Ophthalmol Sep 2009; 127: 1115–1128. doi:10.1001/archophthalmol.2009.233 19752420 PMC2806600

