# Supplementary material for: Investigating ocular ischemic events following pars plana vitrectomy
Source: Eur J Ophthalmol. 2025 Dec 11;36(3):745–54. doi: 10.1177/11206721251403004 (PMC13091923; doi:10.1177/11206721251403004)
Supplement: sj-docx-1-ejo-10.1177_11206721251403004 - Supplemental material for Investigating ocular ischemic events following pars plana vitrectomy [file sj-docx-1-ejo-10.1177_11206721251403004.docx]

**Supplemental Table 1.** Incident cases of RAO, RVO, NAION, and overall vascular occlusive events following vitrectomy by postoperative interval, with percent of total cases.

| **Time Interval After Vitrectomy** | **Number of RAO (% of Total Cases)** | **Number of RVO**  **(% of Total Cases)** | **Number of NAION**  **(% of Total Cases)** | **Number of overall vascular occlusion (% of Total Cases)** |
| --- | --- | --- | --- | --- |
| 0-15 days | 70 (25.3%) | 222 (29.1%) | 27 (22.0%) | 319 (27.5%) |
| 16-30 days | 14 (5.1%) | 37 (4.9%) | 8 (6.5%) | 59 (5.1%) |
| 2 months | 13 (4.7%) | 55 (7.2%) | 11 (8.9%) | 79 (6.8%) |
| 3 months | 16 (5.8%) | 45 (5.9%) | 9 (7.3%) | 70 (6.0%) |
| 4 months | 15 (5.4%) | 45 (5.9%) | 6 (4.9%) | 66 (5.7%) |
| 5 months | 10 (3.6%) | 27 (3.5%) | 4 (3.3%) | 41 (3.5%) |
| 6 months | 15 (5.4%) | 26 (3.4%) | 10 (8.1%) | 51 (4.4%) |
| 7 months | 7 (2.5%) | 34 (4.5%) | 3 (2.4%) | 44 (3.8%) |
| 8 months | 8 (2.9%) | 14 (1.8%) | 3 (2.4%) | 25 (2.2%) |
| 9 months | 9 (3.2%) | 26 (3.4%) | 4 (3.3%) | 39 (3.4%) |
| 10 months | 11 (4.0%) | 27 (3.5%) | 4 (3.3%) | 42 (3.6%) |
| 11 months | 8 (2.9%) | 10 (1.3%) | 4 (3.3%) | 22 (1.9%) |
| 12 months | 9 (3.2%) | 25 (3.3%) | 6 (4.9%) | 40 (3.4%) |
| 13 months | 7 (2.5%) | 17 (2.2%) | 1 (0.8%) | 25 (2.2%) |
| 14 months | 5 (1.8%) | 14 (1.8%) | 2 (1.6%) | 21 (1.8%) |
| 15 months | 7 (2.5%) | 22 (2.9%) | 1 (0.8%) | 30 (2.6%) |
| 16 months | 8 (2.9%) | 15 (2.0%) | 2 (1.6%) | 25 (2.2%) |
| 17 months | 5 (1.8%) | 15 (2.0%) | 2 (1.6%) | 22 (1.9%) |
| 18 months | 12 (4.3%) | 19 (2.5%) | 1 (0.8%) | 32 (2.8%) |
| 19 months | 7 (2.5%) | 16 (2.1%) | 2 (1.6%) | 25 (2.2%) |
| 20 months | 4 (1.4%) | 8 (1.0%) | 1 (0.8%) | 13 (1.1%) |
| 21 months | 6 (2.2%) | 5 (0.7%) | 3 (2.4%) | 14 (1.2%) |
| 22 months | 3 (1.1%) | 15 (2.0%) | 2 (1.6%) | 20 (1.7%) |
| 23 months | 3 (1.1%) | 10 (1.3%) | 5 (4.1%) | 18 (1.5%) |
| 24 months | 5 (1.8%) | 13 (1.7%) | 2 (1.6%) | 20 (1.7%) |

Abbreviations: RAO, retinal artery occlusion; RVO, retinal vein occlusion; NAION, non-arteritic ischemic optic neuropathy.
