# Supplementary material for: Investigating ocular ischemic events following pars plana vitrectomy
Source: Eur J Ophthalmol. 2025 Dec 11;36(3):745–54. doi: 10.1177/11206721251403004 (PMC13091923; doi:10.1177/11206721251403004)
Supplement: sj-docx-2-ejo-10.1177_11206721251403004 - Supplemental material for Investigating ocular ischemic events following pars plana vitrectomy [file sj-docx-2-ejo-10.1177_11206721251403004.docx]

**Supplemental Table 2.** Rate of Ocular Ischemic Events Following Vitrectomy per 10,000 Vitrectomy Surgeries per Month, by Post-operative Time Interval

| **Time Interval After Vitrectomy** | **Rate of RAO** | **Rate of RVO** | **Rate of NAION** | **Rate of Overall Vascular Occlusion** |
| --- | --- | --- | --- | --- |
| 0 – 1 month | 6.98 | 21.52 | 2.91 | 31.41 |
| 1 – 3 months | 1.2 | 4.15 | 0.83 | 6.19 |
| 3 – 6 months | 1.11 | 2.71 | 0.55 | 4.38 |
| 6 – 12 months | 0.72 | 1.88 | 0.33 | 2.94 |
| 12 – 24 months | 0.5 | 1.17 | 0.17 | 1.83 |

Abbreviations: RAO, retinal artery occlusion; RVO, retinal vein occlusion; NAION, non-arteritic ischemic optic neuropathy.
